# Crystal structure of *O*-ethyl *N*-(eth­oxy­carbon­yl)thio­carbamate

**DOI:** 10.1107/S2056989015016989

**Published:** 2015-09-26

**Authors:** Matthew J. Henley, Alex M. Schrader, Victor G. Young, George Barany

**Affiliations:** a Department of Chemistry, University of Minnesota, Minneapolis, MN 55455, USA

**Keywords:** crystal structure, thio­carbamate, hydrogen bonding

## Abstract

The title compound, C_6_H_11_NO_3_S, provides entries to novel carbamoyl disulfanes and related compounds of inter­est to our laboratory. The atoms of the central O(C=S)N(C=O)O fragment have an r.m.s. deviation of 0.1077 Å from the respective least-squares plane. While several conformational orientations are conceivable, the crystal structure shows only the one in which the carbonyl and the thio­carbonyl moieties are *anti* to each other across the central conjugated C—N—C moiety. Pairs of 2.54 Å N—H⋯S=C hydrogen bonds between adjacent mol­ecules form centrosymmetric dimers in the crystal.

## Related literature   

A variety of methods to prepare the title compound and/or similar structures have been reported; see Delitsch (1874 ▸); Atkins *et al.* (1973 ▸); Barany (1977 ▸, and references therein); Vallejos *et al.* (2009 ▸, and references therein); Barany *et al.* (2015 ▸, and references therein). For closely related structures, see CSD (Groom & Allen, 2014 ▸) refcodes: BORBOA (Vallejos *et al.*, 2009 ▸); GAPPAQ (Kang *et al.*, 2012 ▸). For applications of the title compound in inter­esting chemistry, see: Atkins *et al.* (1973 ▸); Barany & Merrifield (1977 ▸); Shen *et al.* (1998 ▸, and references therein); Barany *et al.* (2006 ▸, 2015 ▸); Vallejos *et al.* (2009 ▸).
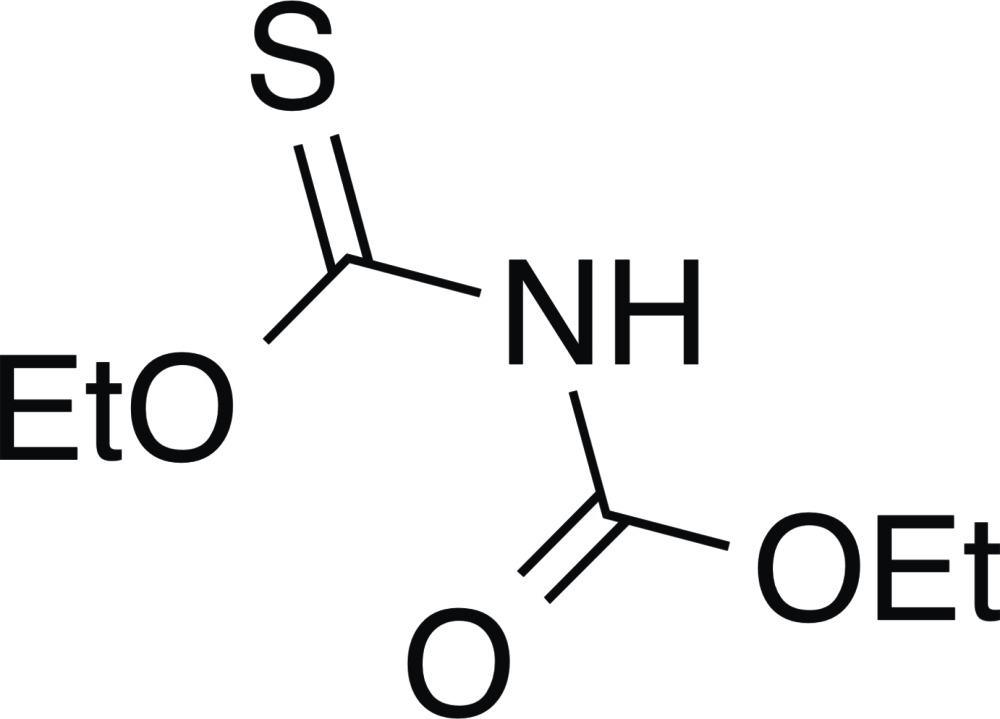


## Experimental   

### Crystal data  

C_6_H_11_NO_3_S
*M* *_r_* = 177.22
Triclinic, 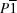

*a* = 4.1782 (17) Å
*b* = 9.236 (4) Å
*c* = 11.820 (5) Å
α = 98.190 (5)°
β = 98.571 (5)°
γ = 102.360 (5)°
*V* = 433.3 (3) Å^3^
*Z* = 2
Mo *K*α radiation
μ = 0.34 mm^−1^
*T* = 173 K
0.50 × 0.10 × 0.05 mm

### Data collection  

Siemens SMART Platform CCD diffractometer
Absorption correction: multi-scan (*SADABS*; Bruker, 2001 ▸) *T*_min_ = 0.851, *T*_max_ = 0.984
4216 measured reflections
1518 independent reflections
1194 reflections with *I* > 2σ(*I*)
*R*_int_ = 0.037

### Refinement  

*R*[*F* ^2^ > 2σ(*F* ^2^)] = 0.040
*wR*(*F* ^2^) = 0.087
*S* = 1.07
1518 reflections
102 parameters
H-atom parameters constrained
Δρ_max_ = 0.25 e Å^−3^
Δρ_min_ = −0.25 e Å^−3^

### 

Data collection: *SMART* (Bruker, 2007 ▸); cell refinement: *SAINT* (Bruker, 2007 ▸); data reduction: *SAINT*; program(s) used to solve structure: *SHELXS97* (Sheldrick, 2008 ▸); program(s) used to refine structure: *SHELXL2014* (Sheldrick, 2015 ▸); molecular graphics: *Mercury* (Macrae *et al.*, 2008 ▸); software used to prepare material for publication: *SHELXTL* (Sheldrick, 2008 ▸).

## Supplementary Material

Crystal structure: contains datablock(s) I, New_Global_Publ_Block. DOI: 10.1107/S2056989015016989/ld2135sup1.cif

Structure factors: contains datablock(s) I. DOI: 10.1107/S2056989015016989/ld2135Isup2.hkl

Supporting information file. DOI: 10.1107/S2056989015016989/ld2135Isup3.cdx

Supporting information file. DOI: 10.1107/S2056989015016989/ld2135Isup4.cml

**Fig. 1.**
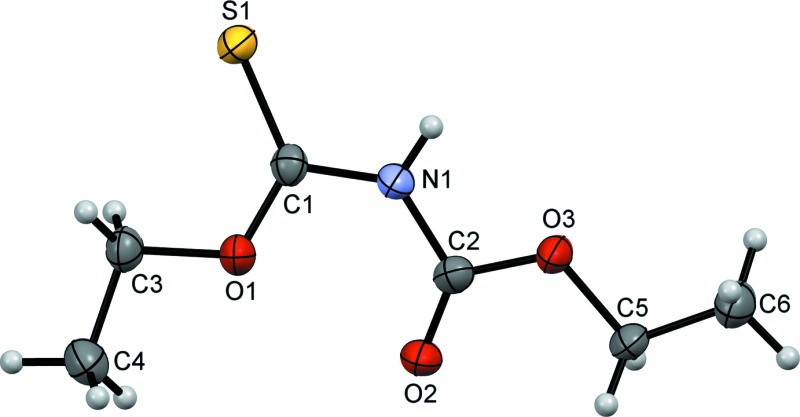
Crystallographic structure of O-ethyl N-(ethoxycarbonyl)thiocarbamate (1) showing 50% probability displacement ellipsoids with all non-hydrogen atoms labeled and numbered.

**Fig. 2.**
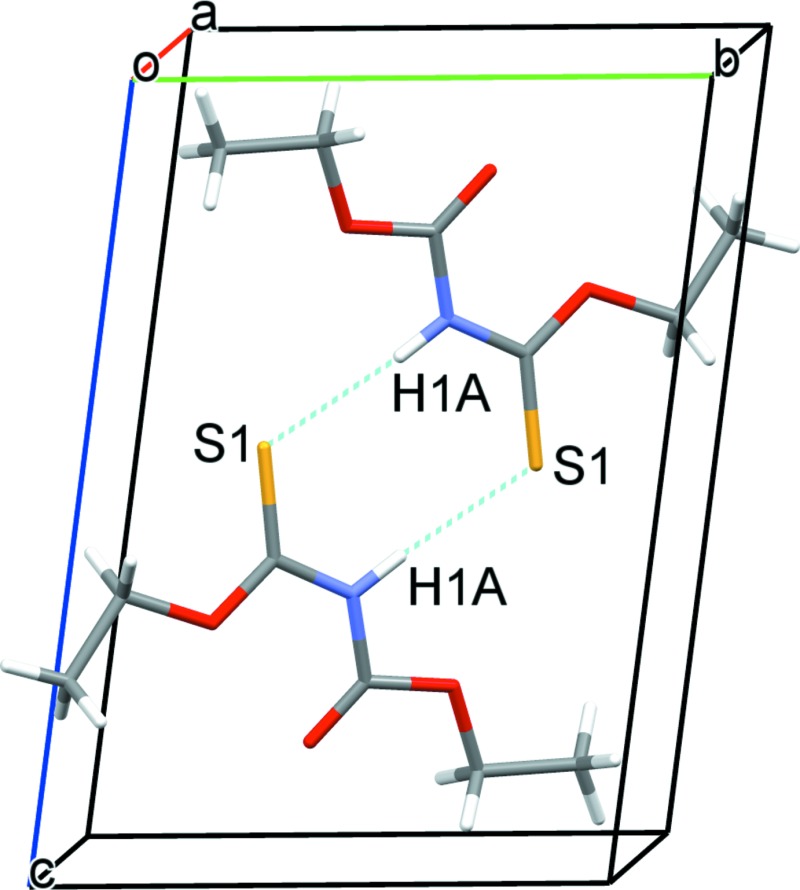
View of crystal packing down the a-axis, with hydrogen bonds highlighted and atoms involved in hydrogen bonding labeled and numbered.

O N 1 . DOI: 10.1107/S2056989015016989/ld2135fig1.tif
Crystallographic structure of *O*-ethyl *N*-(eth­oxy­carbonyl)thio­carbamate (**1**) showing 50% probability displacement ellipsoids with all non-hydrogen atoms labeled and numbered.

a . DOI: 10.1107/S2056989015016989/ld2135fig2.tif
View of crystal packing down the *a*-axis, with hydrogen bonds highlighted and atoms involved in hydrogen bonding labeled and numbered.

CCDC reference: 1423537

Additional supporting information:  crystallographic information; 3D view; checkCIF report

## Figures and Tables

**Table 1:** Hydrogen-bond geometry (, )

*D*H*A*	*D*H	H*A*	*D* *A*	*D*H*A*
N1H1*A*S1^i^	0.88	2.54	3.388(2)	161

Symmetry code: (i) 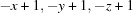.

## supplementary crystallographic information

### S1. Structural commentary

The title compound, *O*-ethyl *N*-(eth­oxy­carbonyl)­thio­carbamate (1), C_6_H_11_NO_3_S, CAS registry # 5585-23-9, also known as (*N*-eth­oxy­thio­carbonyl)­urethane, was of inter­est to a multifaceted organosulfur research program that includes among its goals the development of amino protecting groups removable under mild conditions (Barany, 1977; Barany & Merrifield; 1977; Barany *et al.*, 2006, Barany *et al.*, 2015). Specifically, we have shown that reaction of 1 with (chloro­carbonyl)­sulfenyl chloride gives rise to the novel (chloro­carbonyl)(*N*-eth­oxy­carbonyl­carbamoyl)disulfane, rather than the expected putative *N*-eth­oxy­carbonyl-1,2,4-di­thia­zolidine-3,5-dione (Barany *et al.*, 2015). Variations in reaction conditions and/or the nature of sulfenyl chlorides that react with 1 result in additional unusual structures which model previously uncharacterized inter­mediates in the mechanisms of reactions that successfully elaborate *N*-alkyl-1,2,4-di­thia­zolidine-3,5-dione heterocycles. Thio­carbamate 1 is also useful as an entry to pharmaceutically relevant heterocycles (Atkins *et al.*, 1973), as an additive in the purification of pyrite (Shen *et al.*, 1998), and as a precursor to compounds of inter­est to the agrochemical industry (Vallejos *et al.*, 2009).

X-ray quality crystals of 1 were readily obtained after bulk purification (recrystallization from hot hexane, about 7 mL/g).

All molecular parameters of 1 are within expected ranges. Ignoring the ethyl groups, the central fragment of 1 has a r.m.s. deviation of 0.1077 Å from the least squares plane [i.e., the plane consisting of atoms O1, C1, S1, N1, C2, O2, O3]. The largest deviation of any torsion angle from 0 or 180° is 11.5 (3)° for C2—N1—C1—O1. Although there are multiple theoretically stable conformations of 1 (Vallejos *et al.*, 2009), the molecule uniquely assumes the conformation where the carbonyl and the thio­carbonyl moieties are *anti* to each other across the central C–N–C moiety.

In the crystal, pairs of inter­molecular N–H···S=C hydrogen bonds between two adjacent molecules form centrosymmetric dimers (see Figure 2).

A search of the Cambridge Structural Database (CSD; Version 5.36, update of November 2014; Groom & Allen, 2014) revealed two similar structures, *O*-benzyl *N*-(eth­oxy­carbonyl)­thio­carbamate (2) (Kang *et al.*, 2012) [i.e., BnO(C=S)NH(C=O)OEt], and *O*-ethyl *N*-(meth­oxy­carbonyl)­thio­carbamate (3) (Vallejos *et al.*, 2009) [i.e., EtO(C=S)NH(C=O)OMe]. Inter­estingly, both 2 and 3 assume the same conformation as 1, where the thio­carbonyl and carbonyl moieties are *anti* to each other across the C–N–C moiety. In all three structures, similar length N–H···S=C hydrogen bonds form between adjacent molecules.

### Crystal data

**Table:**

C_6_H_11_NO_3_S	*Z* = 2
*M**_r_* = 177.22	*F*(000) = 188
Triclinic, *P*1	*D*_x_ = 1.358 Mg m^−^^3^
*a* = 4.1782 (17) Å	Mo *K*α radiation, λ = 0.71073 Å
*b* = 9.236 (4) Å	Cell parameters from 1518 reflections
*c* = 11.820 (5) Å	θ = 2.3–25.1°
α = 98.190 (5)°	µ = 0.34 mm^−^^1^
β = 98.571 (5)°	*T* = 173 K
γ = 102.360 (5)°	Thin plates, colorless
*V* = 433.3 (3) Å^3^	0.50 × 0.10 × 0.05 mm

### Data collection

**Table:**

Siemens SMART Platform CCD diffractometer	1518 independent reflections
Radiation source: normal-focus sealed tube	1194 reflections with *I* > 2σ(*I*)
Graphite monochromator	*R*_int_ = 0.037
area detector, ω scans per phi	θ_max_ = 25.1°, θ_min_ = 1.8°
Absorption correction: multi-scan (*SADABS*; Bruker, 2001)	*h* = −4→4
*T*_min_ = 0.851, *T*_max_ = 0.984	*k* = −10→11
4216 measured reflections	*l* = −14→14

### Refinement

**Table:**

Refinement on *F*^2^	0 restraints
Least-squares matrix: full	Hydrogen site location: inferred from neighbouring sites
*R*[*F*^2^ > 2σ(*F*^2^)] = 0.040	H-atom parameters constrained
*wR*(*F*^2^) = 0.087	*w* = 1/[σ^2^(*F*_o_^2^) + (0.0343*P*)^2^ + 0.149*P*] where *P* = (*F*_o_^2^ + 2*F*_c_^2^)/3
*S* = 1.07	(Δ/σ)_max_ < 0.001
1518 reflections	Δρ_max_ = 0.25 e Å^−^^3^
102 parameters	Δρ_min_ = −0.24 e Å^−^^3^

### Special details

**Table:**

Geometry. All e.s.d.'s (except the e.s.d. in the dihedral angle between two l.s. planes) are estimated using the full covariance matrix. The cell e.s.d.'s are taken into account individually in the estimation of e.s.d.'s in distances, angles and torsion angles; correlations between e.s.d.'s in cell parameters are only used when they are defined by crystal symmetry. An approximate (isotropic) treatment of cell e.s.d.'s is used for estimating e.s.d.'s involving l.s. planes.

### Fractional atomic coordinates and isotropic or equivalent isotropic displacement parameters (Å^2^)

**Table:**

	*x*	*y*	*z*	*U*_iso_*/*U*_eq_	
S1	0.08610 (15)	0.30032 (7)	0.46293 (5)	0.0321 (2)	
O1	0.0371 (4)	0.25383 (16)	0.67694 (13)	0.0305 (4)	
O2	0.5131 (4)	0.40638 (17)	0.85405 (13)	0.0346 (4)	
O3	0.8025 (4)	0.61521 (17)	0.80409 (13)	0.0331 (4)	
N1	0.4306 (5)	0.4541 (2)	0.66587 (16)	0.0300 (5)	
H1A	0.5091	0.5211	0.6247	0.036*	
C1	0.1822 (5)	0.3340 (2)	0.60663 (19)	0.0254 (5)	
C2	0.5755 (6)	0.4835 (3)	0.78377 (19)	0.0273 (5)	
C3	−0.2164 (6)	0.1158 (2)	0.6252 (2)	0.0304 (6)	
H3A	−0.1188	0.0435	0.5797	0.037*	
H3B	−0.4004	0.1381	0.5728	0.037*	
C4	−0.3422 (6)	0.0515 (3)	0.7242 (2)	0.0402 (6)	
H4A	−0.5151	−0.0417	0.6936	0.060*	
H4B	−0.4363	0.1245	0.7687	0.060*	
H4C	−0.1576	0.0295	0.7750	0.060*	
C5	0.9980 (6)	0.6597 (3)	0.92221 (18)	0.0301 (6)	
H5A	1.1317	0.5862	0.9389	0.036*	
H5B	0.8494	0.6647	0.9798	0.036*	
C6	1.2223 (7)	0.8123 (3)	0.9279 (2)	0.0413 (7)	
H6A	1.3678	0.8447	1.0045	0.062*	
H6B	1.0865	0.8850	0.9153	0.062*	
H6C	1.3588	0.8068	0.8675	0.062*	

### Atomic displacement parameters (Å^2^)

**Table:**

	*U*^11^	*U*^22^	*U*^33^	*U*^12^	*U*^13^	*U*^23^
S1	0.0366 (4)	0.0312 (4)	0.0232 (3)	0.0006 (3)	−0.0001 (2)	0.0045 (2)
O1	0.0307 (9)	0.0285 (9)	0.0258 (9)	−0.0040 (7)	0.0025 (7)	0.0037 (7)
O2	0.0404 (10)	0.0341 (9)	0.0241 (9)	−0.0032 (8)	0.0031 (7)	0.0103 (8)
O3	0.0388 (10)	0.0286 (9)	0.0228 (8)	−0.0064 (8)	−0.0022 (7)	0.0054 (7)
N1	0.0348 (11)	0.0271 (10)	0.0229 (10)	−0.0034 (9)	0.0018 (9)	0.0082 (8)
C1	0.0242 (12)	0.0229 (12)	0.0281 (12)	0.0053 (10)	0.0024 (10)	0.0048 (10)
C2	0.0302 (13)	0.0290 (13)	0.0222 (12)	0.0064 (11)	0.0056 (10)	0.0037 (10)
C3	0.0296 (13)	0.0245 (12)	0.0325 (13)	0.0004 (10)	0.0023 (11)	0.0027 (10)
C4	0.0386 (15)	0.0377 (15)	0.0406 (15)	−0.0014 (12)	0.0064 (12)	0.0121 (12)
C5	0.0317 (13)	0.0327 (13)	0.0202 (12)	0.0004 (11)	−0.0021 (10)	0.0047 (10)
C6	0.0447 (15)	0.0356 (15)	0.0337 (14)	−0.0040 (12)	−0.0033 (12)	0.0055 (11)

### Geometric parameters (Å, º)

**Table:**

S1—C1	1.654 (2)	C3—H3B	0.9900
O1—C1	1.319 (3)	C4—H4A	0.9800
O1—C3	1.459 (3)	C4—H4B	0.9800
O2—C2	1.191 (3)	C4—H4C	0.9800
O3—C2	1.338 (3)	C5—C6	1.503 (3)
O3—C5	1.462 (3)	C5—H5A	0.9900
N1—C1	1.366 (3)	C5—H5B	0.9900
N1—C2	1.397 (3)	C6—H6A	0.9800
N1—H1A	0.8800	C6—H6B	0.9800
C3—C4	1.498 (3)	C6—H6C	0.9800
C3—H3A	0.9900		
			
C1—O1—C3	118.15 (17)	C3—C4—H4B	109.5
C2—O3—C5	115.13 (16)	H4A—C4—H4B	109.5
C1—N1—C2	128.02 (19)	C3—C4—H4C	109.5
C1—N1—H1A	116.0	H4A—C4—H4C	109.5
C2—N1—H1A	116.0	H4B—C4—H4C	109.5
O1—C1—N1	112.26 (19)	O3—C5—C6	106.43 (18)
O1—C1—S1	126.16 (16)	O3—C5—H5A	110.4
N1—C1—S1	121.57 (17)	C6—C5—H5A	110.4
O2—C2—O3	125.7 (2)	O3—C5—H5B	110.4
O2—C2—N1	127.0 (2)	C6—C5—H5B	110.4
O3—C2—N1	107.34 (18)	H5A—C5—H5B	108.6
O1—C3—C4	106.44 (18)	C5—C6—H6A	109.5
O1—C3—H3A	110.4	C5—C6—H6B	109.5
C4—C3—H3A	110.4	H6A—C6—H6B	109.5
O1—C3—H3B	110.4	C5—C6—H6C	109.5
C4—C3—H3B	110.4	H6A—C6—H6C	109.5
H3A—C3—H3B	108.6	H6B—C6—H6C	109.5
C3—C4—H4A	109.5		
			
C3—O1—C1—N1	−175.56 (18)	C5—O3—C2—N1	−175.86 (18)
C3—O1—C1—S1	4.8 (3)	C1—N1—C2—O2	2.0 (4)
C2—N1—C1—O1	11.6 (3)	C1—N1—C2—O3	−178.5 (2)
C2—N1—C1—S1	−168.77 (18)	C1—O1—C3—C4	−178.52 (19)
C5—O3—C2—O2	3.6 (3)	C2—O3—C5—C6	−177.19 (19)

### Hydrogen-bond geometry (Å, º)

**Table:**

*D*—H···*A*	*D*—H	H···*A*	*D*···*A*	*D*—H···*A*
N1—H1*A*···S1^i^	0.88	2.54	3.388 (2)	161

Symmetry code: (i) −*x*+1, −*y*+1, −*z*+1.
