# Supplementary figures and images for: Crystal structure of O-ethyl N-(eth­oxy­carbon­yl)thio­carbamate
Source: Acta Crystallogr E Crystallogr Commun. 2015 Sep 26;71(Pt 10):o782–3. doi: 10.1107/S2056989015016989 (PMC4647380; doi:10.1107/S2056989015016989)

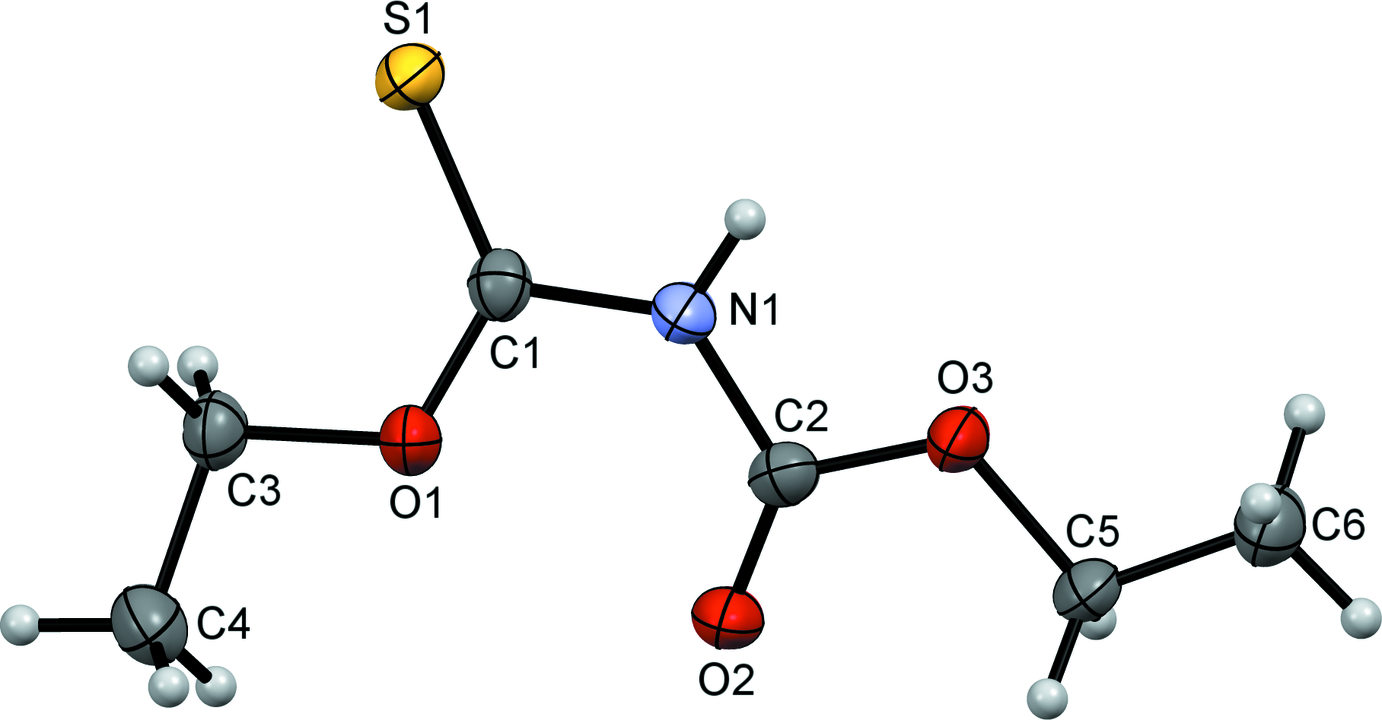

Supplement: Supplementary file 5 [file e-71-0o782-fig1.tif]

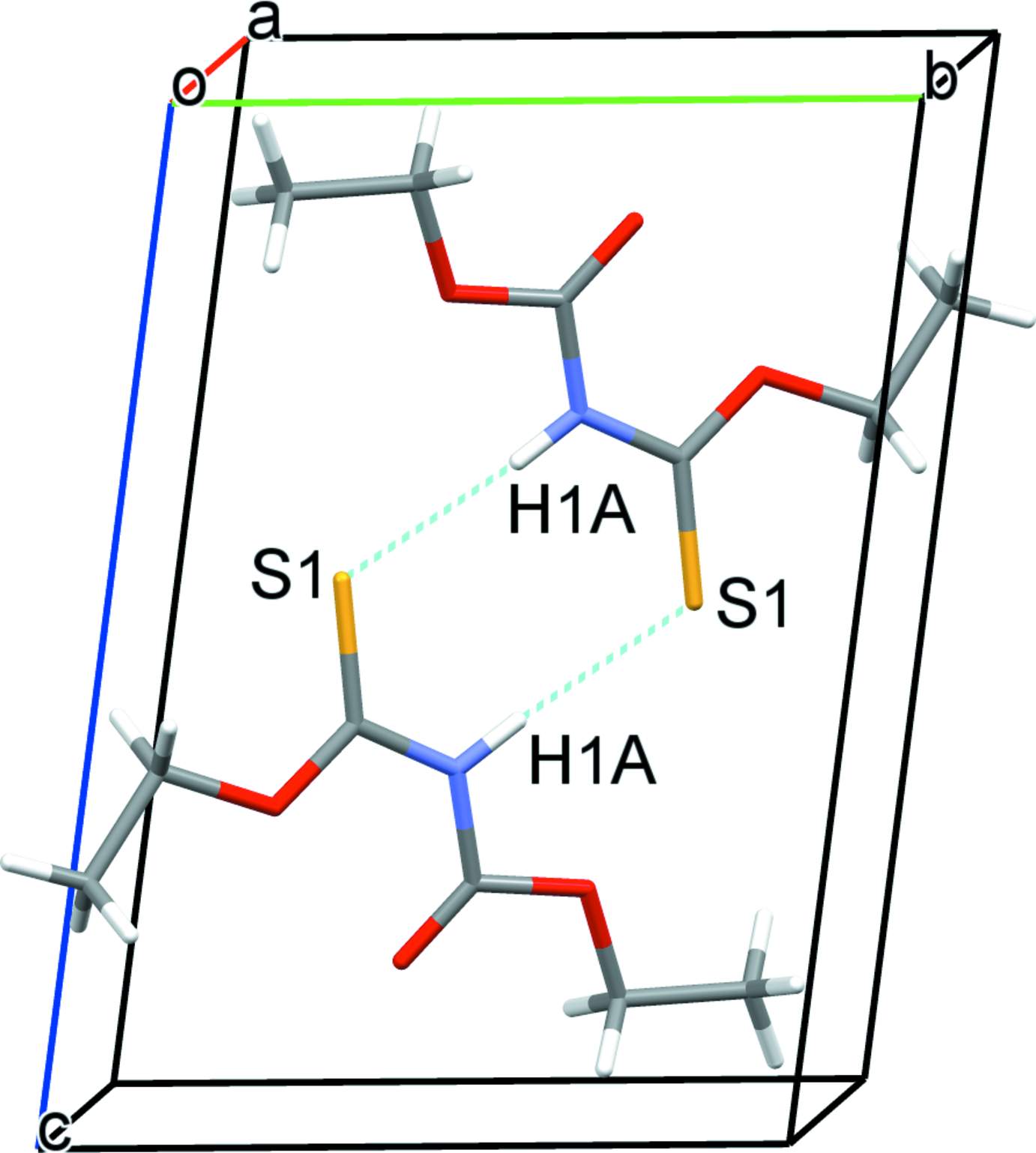

Supplement: Supplementary file 6 [file e-71-0o782-fig2.tif]
